# Endoscopic Ultrasound-Guided Biliary Drainage Using Self-Expandable Metal Stent for Malignant Biliary Obstruction

**DOI:** 10.1155/2017/6284094

**Published:** 2017-04-04

**Authors:** Lei Lu, Xiaowei Tang, Hangbin Jin, Jianfeng Yang, Xiaofeng Zhang

**Affiliations:** Hangzhou First People's Hospital, Nanjing Medical University, Zhejiang, China

## Abstract

*Purpose.* Endoscopic ultrasound-guided biliary drainage (EUS-BD) has been increasingly reported worldwide. However, studies concerning EUS-BD from Mainland China are sporadic. This study aims to investigate the feasibility, efficacy, and safety of EUS-BD using SEMS in a single center from Mainland China. *Methods.* Between November 2011 and August 2015, 24 patients underwent EUS-BD using a standardized algorithm. *Results.* Three patients underwent rendezvous technique (RV), 4 underwent hepaticogastrostomy (HGS), and 17 underwent choledochoduodenostomy (CDS). The technical and clinical success rates were 95.8% (23/24) and 100% (23/23), respectively. Mean procedure time for the CDS group (35.9 ± 5.0 min) or HGS group (39.3 ± 5.0 min) was significantly shorter than that for the RV group (64.7 ± 9.1 min) (*P* < 0.05). Complications (13%) included (1) cholangitis and (2) postprocedure hemorrhage. During the follow-up periods (mean 6.4 months), 22 (91.7%) patients died of tumor progression with mean stent patency of 5.8 ± 2.2 months. Stent occlusion occurred in 2 (8.7%) patients. *Conclusion.* EUS-BD using SEMS is a feasible, effective, and safe alternative for biliary decompression after failed ERCP. EUS-RV may not be the first-line choice for EUS-BD in a medium volume center. Further evaluation and experience of this method are needed.

## 1. Introduction

Endoscopic retrograde cholangiopancreatography (ERCP) is a well-established procedure for the management of malignant biliary obstruction [1–3]. However, even in expert hands, ERCP fails in 3%–5% of cases, especially in patients with surgically altered anatomy or difficult biliary cannulation [4, 5]. Percutaneous transhepatic biliary drainage (PTBD) or surgical bypass has been selected as the salvage procedure in such circumstances. However, PTBD cannot be utilized in the presence of a large amount of ascites and is associated with significant morbidity, such as bile leakage, bleeding, and pneumothorax, and involves uncomfortable external drainage [6]. Surgical bypass is rarely performed because of its significant risk of postoperative morbidity and mortality, especially in patients with advanced malignant biliary obstruction [7].

EUS-guided biliary drainage (EUS-BD) has emerged since the early 2000s as a rescue procedure for patients who fail conventional ERCP [8]. There has been growing global experience with EUS-BD in recent years, and data from expert centers support the feasibility and efficacy of EUS-BD [9–13]. However, few accessories and devices are tailored specifically for this procedure, which limit its development and wide application. Stent selection is a crucial aspect of EUS-BD. Plastic stents were initially used for EUS-BD but similar to their transpapillary biliary application, there were concerns regarding duration of stent patency [9, 14]. This led to the introduction of self-expandable metal stents (SEMS), especially the fully covered SEMS (FCSEMS) [9]. FCSEMS is characteristic by better biliary drainage, prolonged stent patency, and easy stent revision, which may be a promising option for EUS-BD [9, 11, 14].

We therefore conducted this study to prospectively investigate the feasibility, efficacy, and safety of EUS-BD using SEMS in patients with malignant biliary obstruction after failed ERCP. To our knowledge, EUS-BD is not yet commonly used in Mainland China.

## 2. Patients and Methods

### 2.1. Patients

From November 2011 to August 2015, a total of 10,283 ERCP procedures were performed in a 1592-bed tertiary referral hospital and 9394 required biliary decompression. Of these 9394 patients, 152 (1.6%) were candidates for alternative techniques for biliary decompression because of failed ERCP. In total, 128 of these patients were referred for PTBD. The remaining 24 patients had malignant obstructive jaundice and were recruited in this study. The inclusion criteria for this study included (i) initial biliary cannulation or bile duct decompression through ERCP which failed because of accompanying duodenal obstruction, periampullary tumor infiltration, and difficult biliary cannulation and (ii) patients who refused PTBD. The exclusion criteria were (i) refusal to participate in the study protocol, (ii) pregnancy, and (iii) patient age younger than 18 years. Written informed consent was obtained from all patients before the procedure. This study was approved by the Ethics Committee of Hangzhou First People's Hospital and conducted in accordance with the Declaration of Helsinki.

### 2.2. Procedures

All EUS procedures were performed using a therapeutic linear array echoendoscope (GF-UCT240; Olympus, Tokyo, Japan) under general anesthesia. A single experienced endoscopist (X.F.Z.; approximately 1200 ERCP and 500 EUS procedures per year) performed all procedures in an interventional endoscopy room with simultaneous EUS and fluoroscopic capability. A standardized algorithm was used in the current study. EUS-guided rendezvous technique (EUS-RV) drainage was initially attempted in patients with an endoscopically accessible ampulla followed by the transluminal technique for a failed rendezvous attempt. As for transluminal biliary drainage, EUS-guided hepaticogastrostomy (EUS-HGS) was performed in patients with proximal biliary obstruction, surgically altered anatomy, or duodenal obstruction, while EUS-guided choledochoduodenostomy (EUS-CDS) was reserved for patients with middle or distal biliary obstruction. Prophylactic broad-spectrum antibiotics were administered intravenously to all patients before the procedure.

### 2.3. EUS-Guided Rendezvous Technique (EUS-RV) (Figure 1)

By using the echoendoscope, the dilated intrahepatic or extrahepatic bile duct was identified from the proximal gastric body or duodenal bulb. Under real-time EUS and Doppler guidance, a 19-gauge needle (Echotip 19A; Cook Endoscopy, Winston Salem, NC, USA) was inserted into the dilated bile duct with access confirmed by aspiration of bile and cholangiogram. A 0.035-inch guidewire (Jagwire, Boston Scientific, Natick, MA, USA) was advanced in an antegrade direction through the site of stenosis and across the papilla. The guidewire was advanced further to form loops within the duodenal lumen in order to reduce the risk of wire dislodgement. The echoendoscope was then switched to a duodenoscope leaving the guidewire in place. A snare was used to grasp the guidewire and pull it back through the working channel of the duodenoscope. Biliary cannulation was performed in the usual manner over the guidewire. Following successful bile duct access, an uncovered SEMS (UCSEMS, WallFlex; Boston Scientific, Natick, MA, USA) was deployed for biliary drainage.

### 2.4. EUS-Guided Transluminal Technique (EUS-HGS or EUS-CDS) (Figures 2 and 3)

After the bile duct was accessed as described earlier, the biliary-enteric fistula was dilated with biliary dilator catheters (Soehendra dilation catheter 6 or 7 Fr; Cook Endoscopy) or cystotome (10 Fr; Wilson-Cook Medical), which acted as a salvage procedure for failed catheter dilation. Finally, an FCSEMS (WallFlex; Boston Scientific, Natick, Mass., USA) was deployed under echoendoscopic and fluoroscopic view.

### 2.5. Definitions

Failed ERCP was defined as failed access to the bile duct despite the use of advanced cannulation techniques. Technique success was defined as the completion of all procedure steps. Clinical success was defined as a decrease in serum bilirubin to less than 50% of the preprocedure value within the first month. Procedure time was defined as the time between the puncture of bile tract and stent placement. Complication was defined as any stent-related complication, including bile leakage, pneumoperitoneum, bleeding, and stent migration. Major complications were defined as those requiring surgical interventions, whereas those that recovered spontaneously or responded to medical therapy or minimally invasive procedures were defined as minor complications. Biliary reintervention was defined as any type of endoscopic, percutaneous, or surgical intervention that was required to improve biliary drainage after stent placement.

### 2.6. Follow-Up

The endpoint of observation was June 2016. Follow-up continued from stent placement to the death of patients or to the end of the study. Patients received telephone follow-up every month after discharge inquiring about complications including abdominal pain, fever, jaundice, or other symptoms, and patients were referred for outpatient or inpatient treatment as indicated. Data collected during follow-up included laboratory studies for liver and kidney functions and abdominal ultrasound exam. Follow-up data were collected prospectively.

### 2.7. Statistical Analysis

Analysis was carried out using the SPSS 23.0 software package (IBM). Results were reported as mean ± standard deviation for quantitative variables and percentages for categorical variables. Continuous variables were analyzed using a *t*-test, and categorical data were compared using the *χ*^2^ test. Cumulative stent patency was analyzed by the Kaplan-Meier method. *P* value less than 0.05 was considered statistically significant.

## 3. Results

### 3.1. Baseline Characteristics

A total of 24 patients were enrolled in the current study. Demographic and clinical characteristics of the patients are summarized in Table 1. The mean age was 64.8 ± 11.4 years, and 54.2% of patients were male. The etiologies of bile duct obstruction included pancreatic cancer (*n* = 9), cholangiocarcinoma (*n* = 5), ampullary cancer (*n* = 5), hilar biliary obstruction caused by metastatic gastric cancer (*n* = 3), metastatic gallbladder cancer (*n* = 1), and distal biliary obstruction caused by metastatic ureteral carcinoma (*n* = 1). Reasons for failed ERCP included duodenal stenosis caused by tumor invasion (*n* = 7), tumor infiltrating papilla (*n* = 7), failed deep biliary cannulation (*n* = 4), a preexisting duodenal stent (*n* = 2), gastric outlet obstruction due to tumor infiltration (*n* = 2), surgically altered anatomy (*n* = 1, Billroth II resection for gastric cancer), and periampullary duodenal diverticulum (*n* = 1).

### 3.2. Technical and Clinical Outcomes

**Figure 4:**
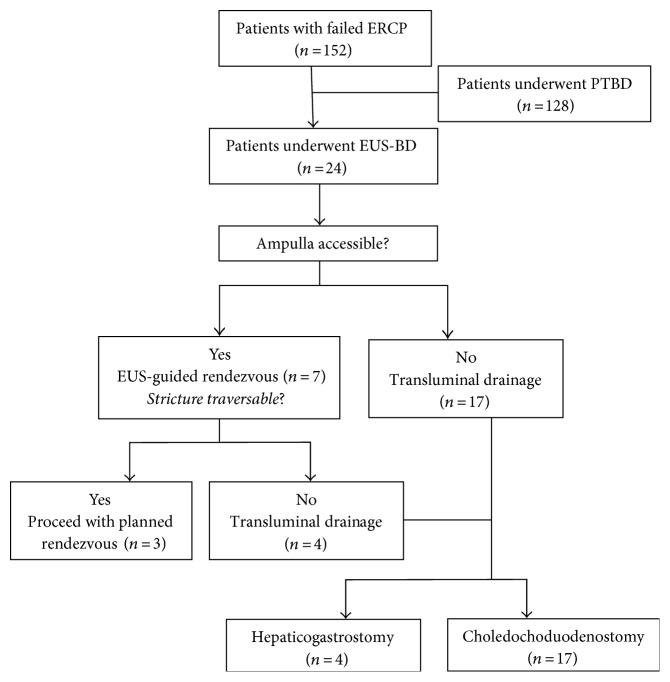
The patient flow diagram.

EUS-BD with stent placement was technically successful in 23 of 24 patients (95.8%), and clinical success was achieved in all patients (23/23, 100%) who had achieved technical success. Details of biliary interventions and outcomes are summarized in Table 2. Of the 23 cases, three underwent EUS-RV, three underwent EUS-HGS, and others underwent EUS-CDS. The patient flow diagram is presented in Figure 4. Obstruction was at the level of the distal bile duct in twenty patients and at the hepatic hilum in four patients. The mean maximum bile duct diameter of those patients with distal biliary obstruction before puncture was 17.4 ± 3.7 mm and 10.0 ± 2.2 mm for patients with proximal biliary obstruction (*P* = 0.013). Mean procedure time for patients who underwent RV was 64.7 ± 9.1 minutes, HGS 39.3 ± 5.0 minutes, and CDS 35.9 ± 5.0 minutes. Mean procedure time for patients who underwent CDS or HGS was significantly shorter than that for patients who underwent RV (*P* < 0.05 for both comparisons). Mean procedure time was not different in patients who underwent CDS with those that underwent HGS (*P* = 0.296).

The failed case was a patient with obstructive jaundice and gastric outlet obstruction. Puncture of the dilated intrahepatic bile duct was achieved, while the hydrophilic guidewire slid out of the intrahepatic biliary system. No procedure-related complications were encountered in this patient. The patient was subsequently referred for PTBD and ultimately had resolution of jaundice.

### 3.3. Complications

Procedure-related complications occurred in three patients (3/23, 13.0%), including one in the HGS group (cholangitis) and two in the CDS group (2 postprocedure hemorrhage), as shown in Table 2. The complications were all minor ones, and no severe complications or procedure-related death was observed. One patient with obstructive jaundice and surgically altered anatomy had transgastric-transhepatic stent placement followed by cholangitis that was managed conservatively with antibiotics. Bleeding was observed in the CDS group. One patient with metastatic ureteral carcinoma had been given low molecular heparin (4400 U/day) four days before the procedure after a normal platelet count, and coagulative tests were confirmed. Bloody stools developed 1 day postoperatively with a decrease in the hemoglobin level (from 12.3 mg/dL to 11.8 mg/dL) and without a need for a blood transfusion. Hemostasis was achieved by endoscopic injection of norepinephrine with clip application, and rebleeding did not occur. Another patient with bilio-enteric tract dilation using cystotome and graded dilation technique developed self-limited bleeding (melena) 2 days postoperatively, which was resolved with conservative treatment.

### 3.4. Follow-Up

**Figure 5:**
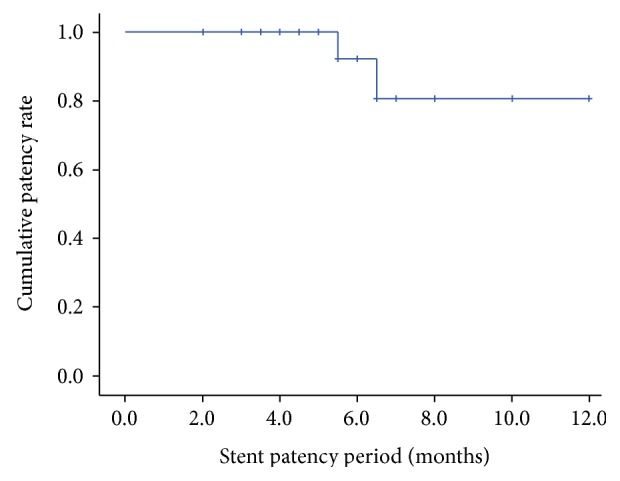
Kaplan-Meier analysis of mean stent patency duration in patients undergoing EUS-BD.

None of the patients were lost during follow-up (mean 6.4 ± 3.1 months). During the follow-up periods, two patients (2/23, 8.7%) presented with stent occlusion because of tumor ingrowth at 5.5 and 6.5 months after EUS-guided interventions, respectively. Two additional procedures were required for successful recanalization, with insertion of a second FCSEMS. The mean duration of stent patency was 5.8 ± 2.2 months (Figure 5). In addition, one patient with a duodenal stent and choledochoduodenostomy had duodenal restenosis by tumor ingrowth 3 months after duodenal stenting. For this particular patient, an additional duodenal stent was inserted. During the follow-up period, 22 patients (22/24, 91.7%) died because of primary cancer progression.

## 4. Discussion

The technologic advances in echoendoscope as well as the close proximity of the transducer to the dilated bile duct have made EUS-BD possible [8, 15]. Since its first description in 2001, EUS-BD has been reported by multiple authors with high success and acceptable complication rate, suggesting it is an effective alternative to PTBD or biliary bypass surgery after failed ERCP [9–13, 16, 17]. In the current study, EUS-BD using SEMS showed satisfactory clinical efficacy with low complication rate, which were consistent with those of previous single-center reports (Table 3) [12, 18–31]. No spontaneous stent migration or bile leakage was observed.

EUS-BD was initially primarily performed using plastic stents, but more recently reported studies have been published with favorable outcomes using FCSEMS. Theoretically, FCSEMS provides several advantages over conventional plastic stents [9, 14]. First, FCSEMS with a larger caliber might afford better drainage and longer stent patency when compared with plastic stents. Second, FCSEMS can decrease the risk of fatal bile leakage and subsequent bile peritonitis by sealing the transmural fistula after stent expansion. Last but not least, FCSEMS can prevent tissue hyperplasia, which makes it easy to be removed endoscopically, especially in patients with benign diseases or needing stent revision because of stent dysfunction. Park et al. [9] in 2009 prospectively evaluated the feasibility and usefulness of EUS-BD using FCSEMS; all enrolled patients achieved technical and functional success, with minor complications occurred in two patients. A retrospective study of 240 patients who underwent EUS-BD by Gupta et al. [32] reported a trend toward better outcomes for metal stents when compared to patients with plastic stent placement, and a significantly higher incidence of cholangitis was observed in patients with plastic stents (11% versus 3%, *P* = 0.02). As for our study, FCSEMS was utilized for transmural drainage. In order to avoid occlusion of the cystic or distal pancreatic duct, UCSEMS was specifically used for rendezvous approach. No bile leakage, pneumoperitoneum, or peritonitis was observed in any of the enrolled patients after the procedure using either the transmural or rendezvous technique. This could be contributed to the application of FCSEMS to some extent. Although stent migration is a worrisome event after FCSEMS placement, the WallFlex stent used in this study is characteristic by strong radial force after stent expansion [33]. Owing to its anchoring effect, no stent migration was observed during the follow-up periods. Recently, a promising lumen-apposing, fully covered self-expandable metal stent has been reported for EUS-guided transmural drainage, and initial reports suggested that it is technically feasible, safe, and effective [34]. However, this type of stent was not available in Mainland China when we conducted this study.

Successful biliary decompression with long-term stent patency is desired. Hara et al. [35] prospectively evaluated the long-term outcomes of EUS-CDS using a plastic stent, a median duration of stent patency of 272 days was presented in their study. A recent prospective multicenter study reported excellent stent patency rates, in which the majority (91%) of patients underwent EUS-BD using SEMS. The 6-month and 1-year stent patency rates were 95% and 86%, respectively, with recurrent biliary obstruction observed in only 5 patients [11]. Comparatively, although using SEMS, a mean stent patency rate of 5.8 months was observed in the current study. This could be explained by the fact that patients enrolled in the current study were complex, all with terminal cancer. In the current study, the majority (91.3%) of stents remained patent at the end of the follow-up period, with only two stent occlusions occurred. The mean follow-up period was merely 6.4 months in the current study, which is obviously less than the minimum duration between patient recruitment and observation endpoint (10 months), but is very close to the mean duration of stent patency. The stent patency may be shortened because of the early arrival of follow-up endpoint, not revealing the potential maximum duration of its own.

EUS-BD is a technically challenging procedure when compared with conventional endoscopic techniques; operator experience and strict selection of patients are highly advisable before EUS-BD is attempted. Will et al. [36] emphasized the importance of expertise in EUS-guided interventions to avoid complications in EUS-BD. Poincloux et al. [20] reported a learning curve effect in their large-scale study; five procedure-related deaths were observed in the first 50 patients during the first 5 years when compared with one death in the last 51 patients during the last 2 years of the study. This has been supported by a Spanish national retrospective study [37]. In the current study, the single technical failure occurred in the first enrolled case and complication rate for the first 2 years was significantly higher than that for the last two years (37.5% versus 0%, *P* = 0.032). The significant morbidity observed at the early stage seems to decrease with the learning curve. However, the small scale of our study may limit the reliability of training effect and further research is warranted.

EUS-BD can be primarily divided into three different techniques: (i) EUS-guided transluminal approach (EUS-HGS and EUS-CDS), (ii) EUS-RV, and (iii) EUS-guided antegrade approach (EUS-AG). Each technique has overlapping indications, and there is currently no consensus for selection of EUS-BD techniques. A theoretical advantage of the rendezvous procedure is the ability to avoid bilio-enteric fistulous tract dilatation and the potential associated risks, particularly bile leakage. However, Khashab et al. [38] suggested that transluminal stenting is comparably safe when biliary decompression is successfully achieved. They compared outcomes of EUS-RV and EUS-transluminal biliary drainage by using a similar treatment algorithm as we performed. Their results suggested that both techniques seem to be equally effective and safe, and EUS-transluminal biliary drainage is a reasonable alternative to EUS-RV. Moreover, Dhir et al. [13] conducted a multicenter study of 68 patients and reported that there was no difference between direct transluminal stenting and EUS-RV regarding efficacy and complications. In the current study, an accessible ampulla was encountered in seven patients and EUS-RV was initially attempted in these patients. However, only three patients (42.9%) ultimately underwent EUS-RV after successful guidewire manipulation. The technique success rate of EUS-RV was lower than that of previous studies, and this could be attributed to limited experience to some extent [39]. Meanwhile, a longer procedure time of EUS-RV was observed in the current study when compared with transluminal drainage. Considering the low technical success rate of rendezvous technique and similar efficacy and safety when compared with transluminal drainage, it seems that EUS-RV is not the first-line choice for a medium volume center with limited experience in EUS-BD.

Our findings must be interpreted with respect to the limitations of this study. First, the number of patients was relatively small, and the follow-up periods were short term, resulting in low statistical power. Second, all procedures were performed by one endoscopist in a single tertiary referral university hospital, and therefore, the results may not be applicable universally. Third, owing to the high mortality observed in the current study, the duration of stent patency and reintervention rate reported in the current study may be underestimated.

In conclusion, in the hands of experienced operators, EUS-BD with SEMS is a feasible, effective, and safe alternative for biliary decompression in patients in whom ERCP was unsuccessful. It should be performed only in tertiary care centers in selected patients. EUS-RV may not be the first-line choice for a medium volume center with limited experience in EUS-BD. A large case series and prospective trials are warranted to further assess this technique.

## Figures and Tables

**Figure 1:**
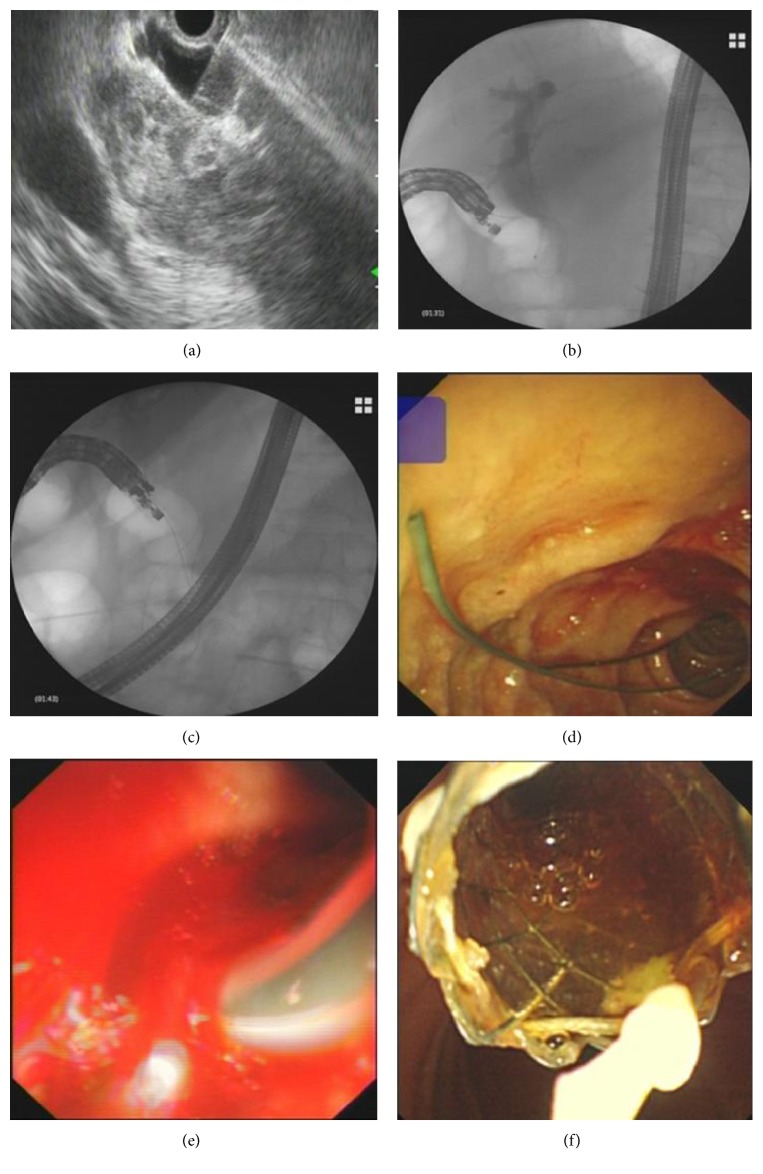
Endoscopic ultrasound-guided rendezvous technique. Under real-time EUS guidance, the dilated bile duct was punctured and a cholangiogram was obtained (a and b). A guidewire was advanced in an antegrade direction through the site of stenosis and across the papilla (c and d). Biliary cannulation was performed in the usual manner over the guidewire (e). Finally, an uncovered self-expandable metal stent was deployed for biliary drainage (f).

**Figure 2:**
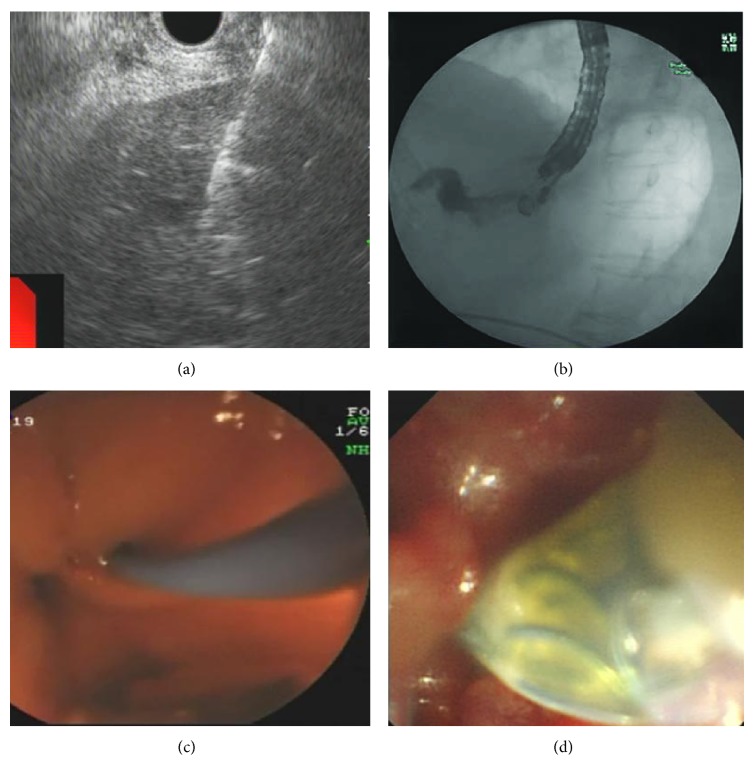
Endoscopic ultrasound-guided hepaticogastrostomy. Under real-time EUS guidance, the dilated intrahepatic bile duct was punctured and a cholangiogram was obtained (a and b). After transmural fistula dilation (c), a fully covered self-expandable metal stent was deployed (d).

**Figure 3:**
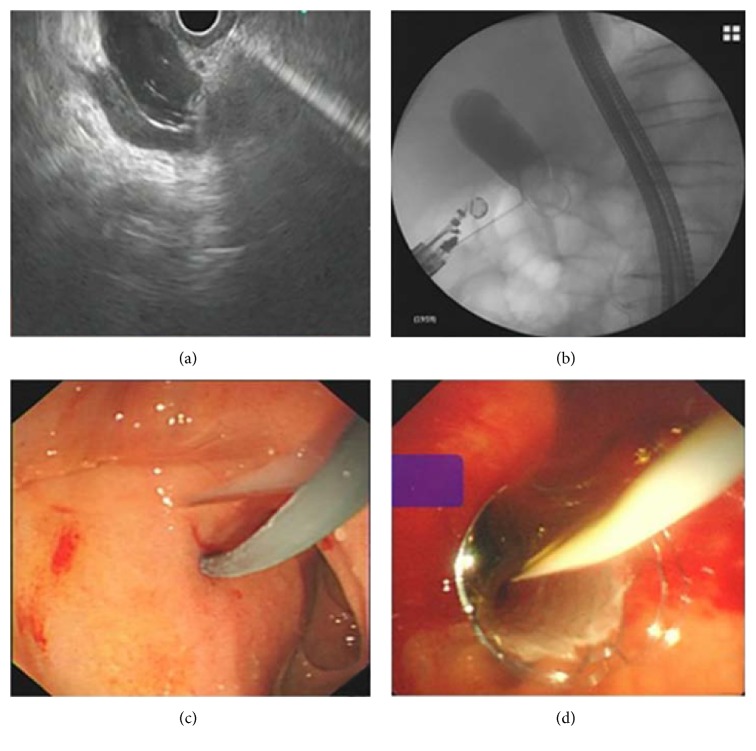
Endoscopic ultrasound-guided choledochoduodenostomy. Under real-time EUS guidance, the dilated extrahepatic bile duct was punctured and a cholangiogram was obtained (a and b). After biliary-enteric fistula dilation (c), a fully covered self-expandable metal stent was deployed (d).

**Table 1:** Demographic and clinical characteristics of the study patients undergoing EUS-BD.

Characteristics	EUS-guided biliary drainage (*n* = 24)
Age, mean (SD), years	64.8 (11.4)
Sex, male, *n* (%)	13 (54.2)
Causes of biliary obstruction, *n* (%)	
Pancreatic cancer	9 (37.5)
Cholangiocarcinoma	5 (20.8)
Ampullary cancer	5 (20.8)
Metastatic gastric cancer	3 (12.5)
Metastatic gallbladder cancer	1 (4.2)
Metastatic ureteral carcinoma	1 (4.2)
Reasons for ERCP failure, *n* (%)	
Duodenal stenosis	7 (29.2)
Tumor infiltrating papilla	7 (29.2)
Failed deep biliary cannulation	4 (16.7)
Preexisting duodenal stent	2 (8.3)
Gastric outlet obstruction	2 (8.3)
Surgically altered anatomy	1 (4.2)
Periampullary duodenal diverticulum	1 (4.2)
Presence of ascites, *n* (%)	4 (16.7)
Previous duodenal stent, *n* (%)	3 (12.5)

**Table 2:** Technique details and follow-up results of EUS-BD.

Outcomes	EUS-guided biliary drainage (*n* = 24)
Successful biliary access, *n* (%)	24 (100)
Technique success, *n* (%)	23 (95.8)
Clinical success, *n* (%)	23 (100)
Type of procedure, *n* (%)	
EUS-HGS	3 (13)
EUS-CDS	17 (74)
EUS-RV	3 (13)
Obstruction site of bile duct, *n* (%)	
Hepatic hilum	4 (16.7)
Distal bile duct	20 (83.3)
Maximum bile duct diameter, mean (SD), mm	
Patients with distal biliary obstruction	17.4 (3.7)^a^
Patients with proximal biliary obstruction	10.0 (2.2)
Procedure time, mean (SD), min	40.1 (11.1)
EUS-HGS	39.3 (5.0)^b^
EUS-CDS	35.9 (5.0)
EUS-RV	64.7 (9.1)^c^
Stent size (diameter and length), *n* (%)	
FCSEMS	20 (87)
8 mm × 6 cm	2 (8.7)
10 mm × 4 cm	2 (8.7)
10 mm × 6 cm	11 (47.8)
10 mm × 8 cm	5 (21.7)
UCSEMS	3 (13)
10 mm × 6 cm	3 (13)
Follow-up period, mean (SD), months	6.4 (3.1)
Stent patency, mean (SD), months	5.8 (2.2)
Complications, *n* (%)	3 (13)
Cholangitis	1 (4.3)
Bleeding	2 (8.7)
Complication rate, % (n/m)	
First 2 years	37.5 (3/8)
Last 2 years	0 (0/15)^d^
Reintervention, *n* (%)	
Stent occlusion	2 (8.7)
Prognosis, *n* (%)	
Dead	22 (91.7)
Alive	2 (8.3)

EUS-RV: endoscopic ultrasound-guided rendezvous technique; EUS-HGS: endoscopic ultrasound-guided hepaticogastrostomy; EUS-CDS: endoscopic ultrasound-guided choledochoduodenostomy. ^a^The mean maximum bile duct diameter of those patients with distal biliary obstruction before puncture (17.4 ± 3.7 mm) was significantly larger than that of patients with proximal biliary obstruction (10.0 ± 2.2 mm) (*P* = 0.013). ^b^There was no significant difference in mean procedure time between the CDS group and HGS group (*P* = 0.296). ^c^Mean procedure time for the CDS group or HGS group was significantly shorter than that for the RV group (*P* < 0.05 for both comparisons). ^d^The complication rate in the first 2 years (37.5%, 3/8) was higher than that for the last two years (0%, 0/15) (*P* = 0.032).

**Table 3:** Summary of previous single-center reports (case number > 20) and current study on EUS-BD using SEMS.

Study	Patients, number	Technique	Stent	Technique success %	Clinical success %	Complication rate %
Cho et al. [18]	54	EUS-HGS/EUS-CDS	PCSEMS	100	94.4	16.6
Bill et al. [19]	25	EUS-RV	PCSEMS	76	96	16
Poincloux et al. [20]	101	EUS-HGS/EUS-CDS/EUS-RV/EUS-CJS	PS/FCSEMS/PCSEMS	98	92.1	11.9
Weilert [21]	21	EUS-AG/EUS-RV/EUS-CDS/EUS-HGS	SEMS/PS	95.2	90.4	9.5
Song et al. [22]	27	EUS-CDS/EUS-HGS	Hybrid metal stent	100	96.3	18.5
Paik et al. [23]	28	EUS-HGS	FCSEMS	96.4	88.9	7.1
Khashab et al. [12]	22	EUS-RV/EUS-CDS/EUS-HGS	SEMS	86.4	86.4	18.2
Artifon et al. [24]	49	EUS-CDS/EUS-HGS	PCSEMS	93.9	84.8	16.3
Takada et al. [25]	32	EUS-AG/EUS-RV/EUS-CDS/EUS-HGS	SEMS	90.6	100	20.7
Prachayakul and Aswakul [26]	21	EUS-CDS/EUS-HGS	FCSEMS	95.2	90.5	9.5
Park et al. [27]	45	EUS-AG/EUS-RV/EUS-CDS/EUS-HGS	FCSEMS/UCSEMS	91	95	11
Attasaranya et al. [28]	31	EUS-CDS/EUS-HGS	PCSEMS/PS	77.4	96	35
Iwashita et al. [29]	40	EUS-RV	SEMS	73	NA	13
Park et al. [30]	57	EUS-CDS/EUS-HGS	FCSEMS/PS	96.5	89	20
Horaguchi et al. [31]	21	EUS-CDS/EUS-HGS/EUS-AG	SEMS	100	NA	14.3
Present study	24	EUS-RV/EUS-CDS/EUS-HGS	FCSEMS/UCSEMS	96	100	13

EUS-BD: endoscopic ultrasound-guided biliary drainage; EUS-HGS: endoscopic ultrasound-guided hepaticogastrostomy; EUS-RV: endoscopic ultrasound-guided rendezvous technique; EUS-CDS: endoscopic ultrasound-guided choledochoduodenostomy; EUS-CJS: endoscopic ultrasound-guided cholangiojejunostomy; SEMS: self-expandable metal stents; PCSEMS: partially covered self-expandable metal stents; FCSEMS: fully covered self-expandable metal stents; UCSEMS: uncovered self-expandable metal stents; PS: plastic stent.
